# Metabolic Dysfunction-Associated Fatty Liver Disease and Fibrosis Status in Patients with Type 2 Diabetes Treated at Internal Medicine Clinics: Türkiye DAHUDER Awareness of Fatty Liver Disease (TR-DAFLD) Study

**DOI:** 10.5152/tjg.2024.24045

**Published:** 2024-08-01

**Authors:** Yasin Şahintürk, Gökhan Köker, Nizameddin Koca, Hilmi Erdem Sümbül, İsmail Demir, Havva Keskin, Selçuk Yaylacı, İhsan Solmaz, Banu Açmaz, Hamit Yıldız, Sibel Ocak Serin, Şükriye Taşçı, Teslime Ayaz, Eşref Araç, Hasan Sözel, Ali Kırık, Attila Önmez, Seher Kır, Hacer Şen, Alihan Oral, Fatih Necip Arıcı, Mustafa Kanat, Ayhan Hilmi Çekin, Seyit Uyar

**Affiliations:** 1 Department of Internal Medicine, University of Health Sciences Antalya Training and Research Hospital, Antalya, Türkiye; 2 Department of Internal Medicine, Bursa City Hospital, Bursa, Türkiye; 3 Department of Internal Medicine, Adana City Hospital, Adana, Türkiye; 4 Department of Internal Medicine, University of Health Sciences Bozyaka Training and Research Hospital, İzmir, Türkiye; 5 Department of Internal Medicine, Ankara University Faculty of Medicine, Ankara, Türkiye; 6 Department of Internal Medicine, Sakarya University Faculty of Medicine, Sakarya, Türkiye; 7 Department of Internal Medicine, University of Health Sciences Diyarbakır Gazi Yaşargil Training and Research Hospital, Diyarbakır, Türkiye; 8 Department of Internal Medicine, Kayseri City Hospital, Kayseri, Türkiye; 9 Department of Internal Medicine, Gaziantep University Faculty of Medicine, Gaziantep, Türkiye; 10 Department of Internal Medicine, University of Health Sciences Ümraniye Training and Research Hospital, İstanbul, Türkiye; 11 Department of Internal Medicine, Karadeniz Technical University Faculty of Medicine, Trabzon, Türkiye; 12 Department of Internal Medicine, Rize University Faculty of Medicine, Rize, Türkiye; 13 Department of Internal Medicine, Dicle University Faculty of Medicine, Diyarbakır, Türkiye; 14 Department of Internal Medicine, Akdeniz University Faculty of Medicine, Antalya, Türkiye; 15 Department of Internal Medicine, Balıkesir University Faculty of Medicine, Balıkesir, Türkiye; 16 Department of Internal Medicine, Düzce University Faculty of Medicine, Düzce, Türkiye; 17 Department of Internal Medicine, Ondokuz Mayıs University Faculty of Medicine, Samsun, Türkiye; 18 Department of Internal Medicine, Biruni University Faculty of Medicine, İstanbul, Türkiye; 19 Department of Internal Medicine, İstanbul Medeniyet University Faculty of Medicine, İstanbul, Türkiye; 20 Department of Gastroenterology, University of Health Sciences Antalya Training and Research Hospital, Antalya, Türkiye

**Keywords:** Type 2 diabetes, metabolic dysfunction-associated fatty liver disease, ultrasound imaging, fibrosis index, awareness, MASLD

## Abstract

**Background/Aims:**

This awareness study aimed to determine the ultrasound (US) examination rates in relation to US-confirmed metabolic dysfunction-associated fatty liver disease (MAFLD) diagnosis in internal medicine outpatients with type 2 diabetes (T2D) across Türkiye.

**Materials and Methods:**

A total of 6283 T2D patients were included in this multicenter retrospective cohort study conducted at 17 internal medicine clinics across Türkiye. The presence and indications for US performed within the last 3 years were recorded along with US-confirmed MAFLD rates, laboratory findings on the day of US, and referral rates. Fibrosis-4 (FIB-4) index was calculated to estimate the risk of advanced liver fibrosis (FIB-4 index ≥ 1.3).

**Results:**

Overall, 1731 (27.6%) of 6283 patients had US examination, which revealed MAFLD diagnosis in 69.9% of cases. In addition, 24.4% of patients with US-confirmed MAFLD were at risk of advanced fibrosis (FIB-4 index ≥ 1.3), and the referral rate was 15.5%.

**Conclusion:**

In conclusion, our findings emphasize an insufficient MAFLD awareness among clinicians and the likelihood of most of T2D patients to be at risk of living with an unknown status regarding their MAFLD and advanced fibrosis risk.

**Main Points**

- Ultrasound (US) examination plus fibrosis-4 (FIB-4) index calculation seems to be a useful method in case-finding for metabolic dysfunction-associated fatty liver disease (MAFLD) and identification of advanced fibrosis risk in internal medicine outpatients with type 2 diabetes (T2D).
- However, this simple imaging-scoring algorithm, despite enabling the diagnosis of MAFLD in ~70% of patients and the risk for advanced fibrosis in ~25% of those with MAFLD, had been applied only in one-third of patients in our cohort.
- The possible underdiagnosis of MAFLD in T2D patients treated at internal medicine clinics seems to indicate that a considerable proportion of T2D patients were living with an unknown status regarding the MAFLD and advanced fibrosis risk.
- Our findings emphasize a need for increased awareness among clinicians on the high prevalence and significant hazards of MAFLD, necessitating its timely diagnosis in T2D patients, and the convenience of US plus FIB-4 index as an easy-to-use strategy in this regard.

## Introduction

Type 2 diabetes (T2D) and fatty liver disease share common pathophysiological mechanisms and their co-existence is mutually detrimental, as each condition increases the development and progression of the other.^1,2^ Non-alcoholic fatty liver disease (NAFLD) refers to fatty infiltration of the liver in the absence of significant alcohol consumption and other chronic liver diseases.^1,3^ Besides its strong link to obesity, T2D, and intestinal microbiome, NAFLD is also regarded as a multisystem disease associated with both liver-related [liver cirrhosis and hepatocellular carcinoma] and extrahepatic [i.e., increased risk of cardiovascular disease and chronic kidney disease complications.^1-4^

Recently, based on the crosstalk between NAFLD and metabolic dysfunction, a change of terminology from NAFLD to metabolic dysfunction-associated fatty liver disease (MAFLD) has been proposed by a panel of international experts, which downplays the importance of alcohol in the definition of NAFLD and emphasizes the metabolic risk factors underlying the disease progression.^5-7^ Accordingly, MAFLD is defined by the presence of fatty liver (hepatic steatosis) plus at least 1 of the 3 criteria, including T2D, overweight/obesity, or evidence of metabolic dysfunction.^5^

Hence, in contrast to NAFLD which is a diagnosis of exclusion, MAFLD diagnosis does not require the exclusion of excessive alcohol consumption or other chronic liver diseases.^2,5^ All T2D patients with hepatic fat content >5% identified by radiological imaging modalities, biological scores with reasonable accuracy or biopsy are considered to have MAFLD.^7,8^ Given the limitations of clinical/laboratory-based risk scores and the invasive nature of liver biopsy, imaging is considered the mainstay tool in the MAFLD diagnosis, while hepatic ultrasound (US) has become the guideline-recommended first-line method for the screening and diagnosis of MAFLD due to widespread availability, relatively low cost, and overall safety.^7-11^

Although there is no universally accepted screening approach for patients at high risk for MAFLD, most guidelines recommend the case-finding (screening) for MAFLD in all high-risk patients (i.e., diabetes, metabolic syndrome, obesity) and agree that US can be useful in screening for MAFLD (in detecting moderate to high levels of steatosis) and also recommend the use of simple scoring systems [i.e., fibrosis-4 (FIB-4) index] in those diagnosed with MAFLD to rule out significant or advanced liver fibrosis.^9-13^

Screening T2D patients for MAFLD is considered a cost-effective strategy, given that T2D patients with concomitant MAFLD represent a highly prevalent and an exceptionally high-risk group within the MAFLD population.^2,7^ However, despite the growing epidemic of MAFLD, in parallel with the epidemics of obesity and diabetes, and the high prevalence and serious clinical implications of MAFLD in patients with T2D, there is limited awareness of and familiarity with the disease among clinicians providing diabetes care.^2,6,8,13-15^

This seems to be the major challenge given the majority of T2D patients with MAFLD are asymptomatic at early stages where internal medicine and endocrinology specialists may play a pivotal role in recognition of the disease as they assess these patients at the frontline.^2,14,15^

In the setting of T2D, presence of MAFLD simply requires the demonstration of >5% hepatic fat without the nuisance of ruling out other chronic liver diseases, which might actually facilitate the diagnosis of the disease by the non-hepatologist.^7,8,16^ Hence, improved awareness of clinicians about the risk and clinical relevance of MAFLD in the setting of T2D is considered to be of utmost importance in fighting this global health challenge, by enabling early identification and appropriate and timely intervention of high-risk MAFLD patients, since even the advanced fibrosis stage is considered potentially reversible upon reversal of the initial injurious stimuli.^2,12^

Therefore, within the context of an awareness-raising project conducted in collaboration with the DAHUDER (Society of Internal Medicine Specialists), this cross-sectional TR-DAFLD (TüRkiye DAHUDER Awareness of Fatty Liver Disease) study aimed to provide a snapshot of the current MAFLD and advanced fibrosis status in a cohort of T2D patients treated at internal medicine clinics across Türkiye, via a simple algorithm based on US imaging and FIB-4 index.

## Materials and Methods

### Study Population

A total of 6283 patients with T2D (mean ± SD age: 57.1 ± 11.9 years, 61.1% were females) for at least 3 years were included in this retrospective multicenter TR-DAFLD study conducted between February 2023 and April 2023 at 17 internal medicine clinics across Türkiye in collaboration with the DAHUDER. T2D patients who presented to internal medicine outpatient clinics for a routine control visit and agreed to participate in the detailed interview performed by the physician during the visit were included in the study on the day of outpatient control visit. Patients with excessive alcohol consumption or other chronic liver diseases were not excluded, given that MAFLD diagnosis does not require the exclusion of these conditions. However, patients with specific liver diseases such as hepatocellular carcinoma, hepatic cirrhosis, and biliary disease were excluded from the study. Although 6297 patients were initially enrolled, 6283 patients comprised the final study population with the exclusion of 14 patients who did not give consent to use their personal data.

Written informed consent was obtained from each subject. The study was conducted in accordance with the ethical principles stated in the Declaration of Helsinki and approved by the institutional ethics committee of Antalya Training and Research Hospital (approval number: 1/11, date: January 12, 2023).

### Assessments

Details on disease background were obtained via history taking, and the acquired information was combined with US findings and laboratory parameters. Overall, patient demographics (age, gender), duration of diabetes, latest glycated hemoglobin (HbA1c) value and the presence of a US examination (including liver parenchyma assessment) performed for any reason within the last 3 years as well as the US-confirmed MAFLD rates were recorded in each patient. In those with US-confirmed MAFLD, the laboratory findings on the day of US and the referral rates (percentage of patients referred to gastroenterology for further investigation) were recorded, while FIB-4 index was also calculated via the following equation: age × aspartate aminotransaminase (AST) [IU/L]/platelet count [ ×100 000/L)] × square root of (alanine aminotransaminase (ALT) [IU/L]). Patients with FIB-4 index ≥1.3 were considered to have the advanced liver fibrosis risk.^17^

### Statistical Analysis

Statistical analysis was performed using the Statistical Package for the Social Sciences® Statistics for Windows, version 25.0 (IBM Corp., Armonk, NY, USA). Descriptive statistics were reported, including mean ± standard deviation, median, interquartile range (IQR), and minimum-maximum values for continuous variables and percentages for categorical variables.

## Results

### Baseline Characteristics

Mean age of patients was 57.1 years (range, 18-99 years), and females comprised 61.1% of the study population. Median duration of diabetes was 9 years (range, 5-13 years) and the latest HbA1c values were 7.6% (range, 6.6-9.2%) (Table 1).

### Ultrasound Examination and Metabolic Dysfunction-Associated Fatty Liver Disease Rates

**Figure 1.**
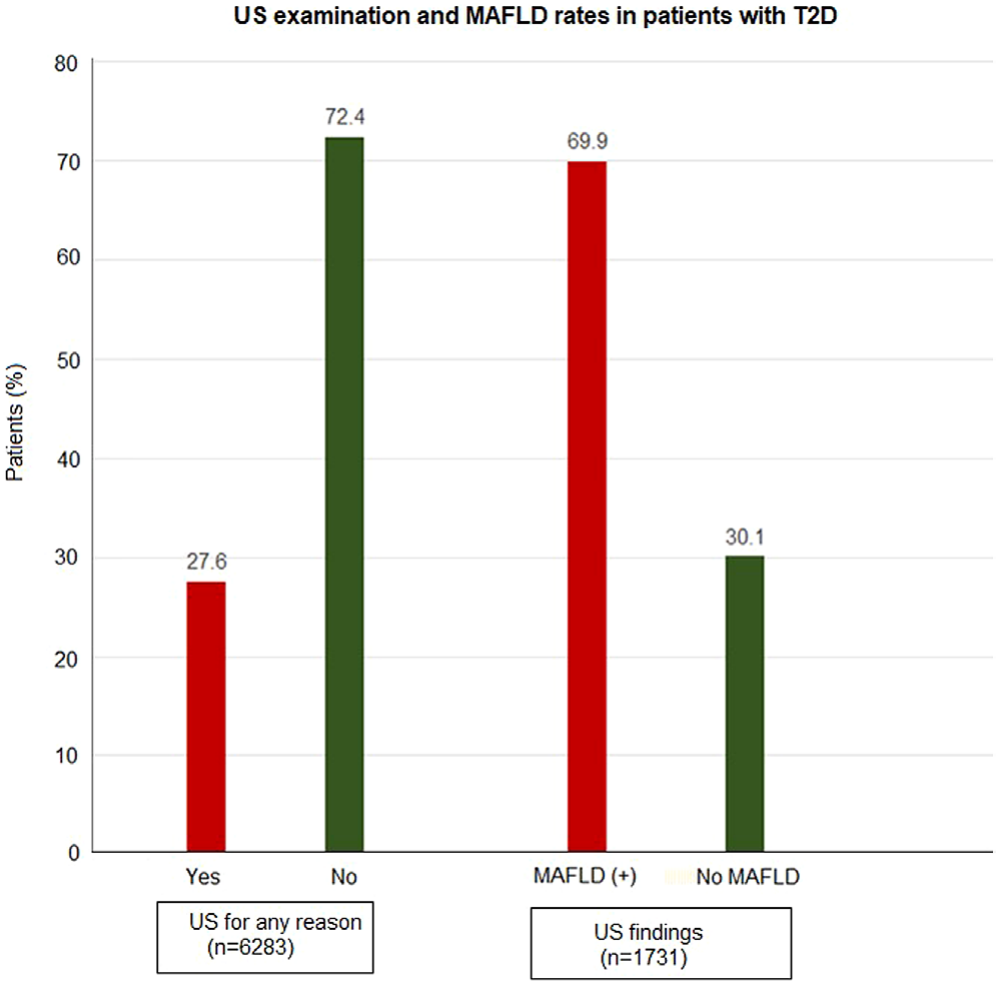
Ultrasound examination and metabolic dysfunction-associated fatty liver disease rates in patients with type 2 diabetes.

**Figure 2.**
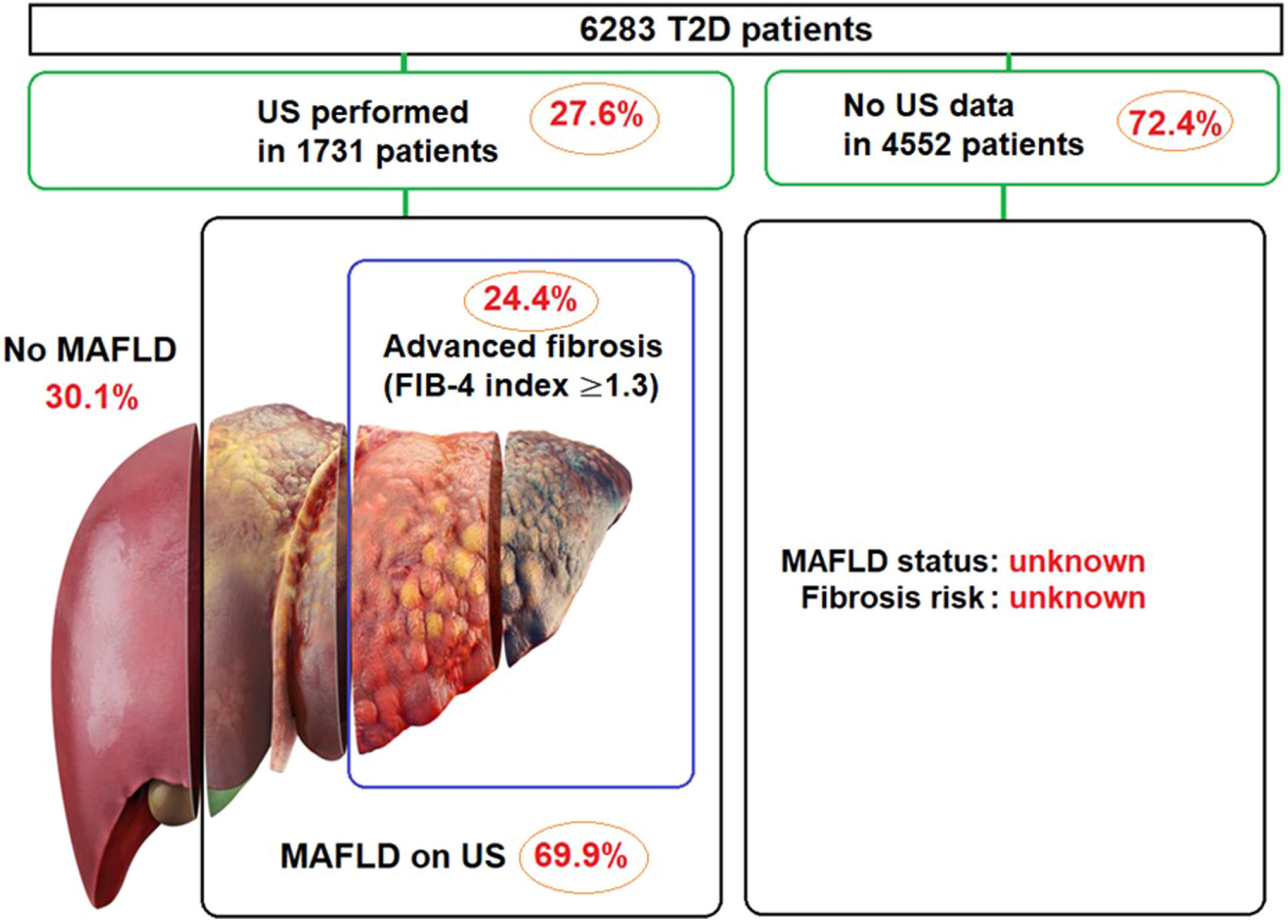
Metabolic dysfunction-associated fatty liver disease; rates and advanced fibrosis status in patients with type 2 diabetes.

Overall, 1731 (27.6%) of 6283 patients were identified to have US examination, and MAFLD was diagnosed in 1211 (69.9%) of these cases. Also, 831 (48.0%) of 1731 US examinations were performed specifically for suspected MAFLD, which revealed the MAFLD diagnosis in 625 (75.2%) cases (Table 1, Figure 1, Figure 2).

### Laboratory Findings in Patients with Ultrasound-Confirmed Metabolic Dysfunction-Associated Fatty Liver Disease

Laboratory findings on the day of US in patients with US-confirmed MAFLD (n = 1211) are summarized in Table 1. Glycated hemoglobin levels were median 7.7% (IQR: 6.7-9.4%), while mean ± SD platelet counts were 284.0 ± 89.0 10^3^/µL. Median (IQR) AST and ALT levels were 21 (16-29) IU/L and 23(16-37) IU/L, respectively.

Median (IQR) FIB-4 index in patients with US-confirmed MAFLD was 0.93 (0.67-1.29), and advanced fibrosis risk (FIB-4 index ≥1.3) was evident in 290 (24.4%) patients (Table 1, Figure 2).

### Referral Rates in Patients with Ultrasound-Confirmed Metabolic Dysfunction-Associated Fatty Liver Disease

Overall, referral for further investigation upon detection of MAFLD on US was performed in 185 (15.5%) of 1190 patients with available data. Referral rates in patients at risk of advanced fibrosis were 17.9% (Table 1).

## Discussion

Our findings in a retrospective cohort of 6283 T2D patients revealed insufficient awareness among internists regarding the screening or case-finding strategy for MAFLD in the setting of T2D. Less than one-third of T2D patients had US examination during their follow-up at internal medicine clinics, which confirmed the presence of MAFLD in 69.9% of cases. Advanced fibrosis risk (FIB-4 index ≥1.3) was evident in 24.4% of patients at the time of US-confirmed MAFLD, while the referral for further investigation was performed in 15.5% of patients.

Türkiye is considered a risky region in terms of NAFLD burden with an estimated 30% prevalence of NAFLD (range, 48.3%-60.1%), which is expected to further increase with rising prevalence of obesity and T2D.^18^ The transabdominal ultrasonography findings from the recent Cappadocia Cohort Study of Türkiye in 2797 subjects (14% with T2D) revealed a high prevalence of hepatic steatosis (60.1%) emphasizing that Türkiye is one of the leading countries in the world for NAFLD.^19^

The rates of US-confirmed MAFLD (69.9%) and advanced fibrosis risk (24.4%) in our patients are in line with consideration of MAFLD to affect over half of T2D patients (up to 75%-90%, possibly), and presence of histological hepatic fibrosis alongside steatosis in approximately 1 in 5 individuals with MALFD.^7,8,16,20^ In a meta-analysis of studies in T2D patients, the global prevalence of MAFLD by US imaging was estimated to be 55.5%, while NASH (i.e., nonalcoholic steatohepatitis) and advanced fibrosis rates on biopsy were 37.3% and 4.8%, respectively.^20^

Nonetheless, despite the high prevalence and significant extra-hepatic complications of MAFLD in T2D patients, it is considered to be usually overlooked in clinical practice.^2,6,8,13-15^ Although most guidelines such as American Association of Clinical Endocrinology and American Association for the Study of Liver Diseases, European Association for the Study of the Liver, European Association for the Study of Diabetes and European Association for the Study of Obesity clinical practice guidelines and World Gastroenterology Organization global guidelines recommend a screening or case-finding strategy for MAFLD for at-risk patients including those with T2D, the implementation of these screening strategies in clinical practice is strongly limited by controversies regarding the diagnostic tests and treatment options for MAFLD.^9-13,21-24^ More importantly, due to low awareness and poor recognition of MAFLD among clinicians, many T2D patients living with MAFLD are considered to be unaware of their fibrosis stage, and those with advanced fibrosis remain at risk of advanced liver disease due to delayed referral to specialists for evaluation and care.^14,25^ Notably, the MAFLD and advanced fibrosis risk findings achieved in our cohort reflect the current status only in one-third of the overall study population, indicating that most patients with T2D had no US examination during their routine follow-up and thus were living with an unknown status regarding the MAFLD and advanced fibrosis risk.

Hence, our findings indicate the possible underdiagnosis of MAFLD in T2D patients treated at internal medicine clinics, emphasizing a need for increased awareness among clinicians regarding the high prevalence of MAFLD and risk of advanced fibrosis in T2D patients, as well as the likelihood of US imaging and FIB-4 index to be used as a simple screening strategy in these patients.

Indeed, as surveillance for liver disease complications is recommended only for patients with severe fibrosis, application of more specific criteria for risk prediction (i.e., FIB-4 and US-determined indices) for referring patients to a hepatologist is considered a cost-effective fatty liver referral pathway, enabling more reasonable referral rates consistent with the underlying advanced fibrosis.^12,21,26^ Otherwise, the process may reveal very high referral rates (33-85%) when referral was applied also for T2D patients with less severe liver disease, despite the physician can continue the standard diabetes care including lifestyle modification in these patients with no need for further referral.^12,21^ In our cohort, with use of these stringent criteria (US plus FIB-4 index), 24.4% of MAFLD patients were found to be at risk of advanced fibrosis (FIB-4 scores ≥3) and the overall referral rate was 15.5%.

The advanced fibrosis risk and referral rates in our study should be interpreted in the light of the possibility of including a larger population of patients at high risk of liver disease progression by definition of MAFLD. The likelihood of underestimating the mild disease in the present study should also be considered, given the exclusion of newly diagnosed T2D patients and the low performance of US for the detection of mild steatosis, since it necessitates the presence of steatosis in at least 12.5%-33% of hepatocytes to detect fatty liver with optimal accuracy.^8,9,20,21^

In a recent study, based on the data from the U.S. National Health and Nutrition Examination Survey in 6727 T2D patients, MAFLD was identified in 4982 patients, which was classified as MAFLD(+)/NAFLD(−) in 2032 patients and MAFLD(+)/NAFLD(+) in 2950 patients.^16^ The new definition (MAFLD) was reported to increase the fatty liver diagnosis in T2D patients by 68.9%, while patients classified as MAFLD(+)/NAFLD(−) were also found to be at a higher risk of major adverse cardiovascular events, advanced fibrosis, all-cause and cardiovascular-related mortality compared to those classified as MAFLD(+)/NAFLD(+).^16^ Accordingly, MAFLD not only identifies more patients due to no exclusion of other chronic liver diseases but also seems to be better in identifying patients at risk of liver and cardiovascular complications, which is considered to indicate a need for better risk stratification to prevent an over-inclusion of fatty liver.^16,27^

Although there are no pharmacological agents approved specifically for treating MAFLD, lifestyle modification, particularly weight reduction via dietary and exercise strategies or bariatric surgery, in addition to statins and some antidiabetic medications (i.e., pioglitazone, glucagon-like peptide 1 receptor agonists and SGLT2 (i.e., sodium-glucose cotransporter-2) inhibitors) with proven benefits in overall improvements in liver histology and hepatic fibrosis are recommended in T2D patients with MAFLD.^2,7,8,10,28,29^ Thus, MAFLD is suggested to be considered an emerging diabetic complication and to be timely diagnosed and systematically evaluated by proactive participation of all health care providers taking care of T2D patients, as in other conventional diabetes-related complications.^2,8,12^

Besides the low awareness among the clinicians on MAFLD, many factors have been implicated in the underdiagnosis of MAFLD in clinical practice, such as the knowledge gaps regarding the risk-factors, diagnosis, and management approaches, the lack of tools to support clinical decision making, and the dearth of national strategies, guidelines, or action plans to address the increasing prevalence of MAFLD.^14,15,21,30-33^ Therefore, improved awareness (via continuing education programs, awareness campaigns, improved guidelines, and referral protocols) among all important stakeholders (primary care physicians, specialists, and health policy makers) is emphasized regarding the addition of MAFLD as another frequent end-organ complication of T2D necessitating timely diagnosis and intervention.^8,14,33-35^

Given that international guidelines increasingly advocate multidisciplinary approaches for patients with MAFLD, the strategies to fight against the underestimation of the disease burden and lack of awareness should also consider the potential interdisciplinary differences in awareness, knowledge and management of MAFLD and thus specifically target the medical specialties where the largest improvements could be made.^23,33,36^

The major strength of this study seems to be the potential generalizability of our results given the inclusion of 6283 T2D patients from 17 internal medicine clinics across Türkiye. However, certain limitations should be considered. First, due to the cross-sectional design, it is impossible to establish any cause-and-effect relationships. Second, since this is an awareness study regarding the US examination and MAFLD diagnosis rates in T2D patients, analysis of patient and treatment characteristics (i.e., family history, concomitant obesity, viral hepatitis, treatment changes in those with MAFLD/advanced fibrosis) was not within the scope of the study. Third, the unknown MAFLD status in most patients due to the absence of US imaging is another potential limitation. Fourth, the exclusion of newly diagnosed T2D patients and the use of US as the sole imaging modality might have resulted in an underestimated diagnosis of mild disease. Nevertheless, this study was conducted in the context of an awareness-raising project to provide a snapshot of the current MAFLD status among T2D patients treated at internal medicine clinics across Türkiye.

In conclusion, our findings revealed the favorable utility of US plus FIB-4 index in case-finding for MAFLD and identification of advanced fibrosis risk with reasonable referral rates in T2D patients treated at internal medicine clinics. However, this simple imaging-scoring algorithm, despite enabling the diagnosis of MAFLD in ~70% of patients and the risk for advanced fibrosis in ~25% of those with MAFLD, had been applied only in one-third of patients and with an indication of suspected MAFLD only in half of them, indicating that most patients with T2D were living with an unknown status regarding the MAFLD and advanced fibrosis risk. Hence, the possible underdiagnosis of MAFLD in T2D patients treated at internal medicine clinics emphasizes a need for increased awareness among clinicians on the high prevalence and significant hazards of MAFLD, necessitating its timely diagnosis in T2D patients, and the convenience of US plus FIB-4 index as an easy-to-use strategy in this regard.

## Figures and Tables

**Table 1.** Baseline Characteristics, Ultrsound Imaging, and Laboratory Findings in Type 2 Diabetes Patients

Demographic and Clinical Characteristics (n = 6283)		
Age (year), mean ± SD (minimum–maximum)		57.1 ± 11.9 (18-99)
Gender, n (%)		
Female		3899 (61.1)
Male		2384 (37.9)
Duration of diabetes (year), median (IQR)		9 (5-13)
Latest HbA1c (%), median (IQR)		7.6 (6.6-9.2)
US imaging, n (%)		
US for any reason (n = 6283)		
Yes		1731 (27.6)
No		4552 (72.4)
US indication (n = 1731)		
For suspected diagnosis of MAFLD		831 (48.0)
For other reasons		900 (52.0)
US-confirmed MAFLD diagnosis (n = 1731)		
Yes		1211 (69.9)
No		520 (30.1)
Laboratory findings on the day of US in patients with MAFLD (n = 121)		
HbA1c (%), median (IQR)		7.7 (6.7-9.4)
AST (IU/L), median (IQR)		21 (16-29)
ALT(IU/L), median (IQR)		23 (16-37)
Platelet count (10^3^/µL), mean ± SD		284.0 ± 89.0
FIB-4 index	median (IQR) (n = 1190)	0.93 (0.67-1.29)
	≥1.3 (advanced fibrosis risk)	290 (24.4)
	<1.3	900 (75.6)
Referral in patients with advanced fibrosis (n = 1190), n (%)		185 (15.5)
(FIB-4 index ≥ 1.3) (n = 290)		52 (17.9)

ALT, alanine aminotransaminase; AST, aspartate aminotransaminase; FIB-4: fibrosis-4; MAFLD, metabolic dysfunction-associated fatty liver disease; US, ultrasound.
